# Cyclophosphamide Alters the Gene Expression Profile in Patients Treated with High Doses Prior to Stem Cell Transplantation

**DOI:** 10.1371/journal.pone.0086619

**Published:** 2014-01-22

**Authors:** Ibrahim El-Serafi, Manuchehr Abedi-Valugerdi, Zuzana Potácová, Parvaneh Afsharian, Jonas Mattsson, Ali Moshfegh, Moustapha Hassan

**Affiliations:** 1 Experimental Cancer Medicine (ECM), Clinical Research Centre (KFC), Department of Laboratory Medicine, Karolinska Institutet, Huddinge, Stockholm, Sweden; 2 Clinical Research Centre (Novum), Karolinska University Hospital-Huddinge, Stockholm, Sweden; 3 Department of Genetics, Royan Institute, Tehran, Iran; 4 Centre for Allogeneic Stem Cell Transplantation, Karolinska University Hospital-Huddinge, Stockholm, Sweden; 5 Department of Therapeutic Immunology, Karolinska Institute, Karolinska University Hospital, Huddinge, Stockholm, Sweden; 6 Cancer Centre of Karolinska (CCK), Department of Oncology-Pathology, Karolinska Institutet, Solna, Stockholm, Sweden; Hospital Infantil Universitario Niño Jesús, Spain

## Abstract

**Background:**

Hematopoietic stem cell transplantation is a curative treatment for several haematological malignancies. However, treatment related morbidity and mortality still is a limiting factor. Cyclophosphamide is widely used in condition regimens either in combination with other chemotherapy or with total body irradiation.

**Methods:**

We present the gene expression profile during cyclophosphamide treatment in 11 patients conditioned with cyclophosphamide for 2 days followed by total body irradiation prior to hematopoietic stem cell transplantation. 299 genes were identified as specific for cyclophosphamide treatment and were arranged into 4 clusters highly down-regulated genes, highly up-regulated genes, early up-regulated but later normalized genes and moderately up-regulated genes.

**Results:**

Cyclophosphamide treatment down-regulated expression of several genes mapped to immune/autoimmune activation and graft rejection including CD3, CD28, CTLA4, MHC II, PRF1, GZMB and IL-2R, and up-regulated immune-related receptor genes, e.g. IL1R2, IL18R1, and FLT3. Moreover, a high and significant expression of ANGPTL1 and c-JUN genes was observed independent of cyclophosphamide treatment.

**Conclusion:**

This is the first investigation to provide significant information about alterations in gene expression following cyclophosphamide treatment that may increase our understanding of the cyclophosphamide mechanism of action and hence, in part, avoid its toxicity. Furthermore, ANGPTL1 remained highly expressed throughout the treatment and, in contrast to several other alkylating agents, cyclophosphamide did not influence c-JUN expression.

## Introduction

Hematopoietic stem cell transplantation (HSCT) is currently used as a curative treatment for a wide range of diseases, including malignancies such as leukaemia and lymphomas and non-malignant diseases such as metabolic and haematological disorders. Conditioning regimens - either a combination of cytostatics or cytostatics together with radiotherapy - are designed to prepare the patient to receive donor stem cells. Cytostatics are administered in order to eliminate malignant cells, provide free space for the donor cells and suppress the immune system in order to prevent graft rejection [1].

Cyclophosphamide (Cy) is an alkylating agent widely used as a part of the conditioning regimen prior to HSCT and for the treatment of haematological malignancies and solid tumours. Cy is used either in combination with other cytostatics or with total body irradiation (TBI) [2]. It acts on DNA by attaching an alkyl group to the guanine base of DNA at the number 7 nitrogen atom of the imidazole ring and leads to guanine-adenine intra-strand cross-linking. This damage to the DNA strand can trigger apoptosis when the cellular machinery fails to repair it.

Cy is also a potent immunosuppressive agent i.e. it is capable of attenuating both humoral and cell-mediated immune responses [3]. Due to these immunosuppressive effects, Cy is used in the treatment of several autoimmune diseases including rheumatoid arthritis and systemic lupus erythematosus [4].

Cy is a prodrug that is metabolized by cytochrome P450 to its main active metabolite, 4-hydroxycyclophosphamide (4-OH-Cy), which comprises about 90% of the total Cy dose [5]. 4-OH-Cy is subsequently converted to phosphoramide mustard and acrolein, which is nephrotoxic. An alternative pathway of Cy transformation is N-dechloroethylation. Moreover, Cy is partly metabolized to the inactive metabolite 2-dechloroethyl-Cy and to chloroacetaldehyde, which is neurotoxic [6]–[8].

Studies on Cy kinetics have shown a high inter-individual variation in elimination half-life and clearance [6], [9]. This variation may be explained by polymorphisms in CYP2B6, the main enzyme responsible for the conversion of Cy to its active form [10]–[12].

Several studies have investigated the clinical efficacy of Cy alone or in combination with other cytostatics or radiotherapy [2], [13]. However, the contribution of Cy to the outcome of HSCT and, more importantly, the mechanisms by which Cy exerts its effect on immune cells has not yet been addressed.

In the past decade, the advent of DNA microarray technology together with the availability of the complete nucleotide sequence of the human genome have allowed elucidation of the molecular mechanisms in several diseases [14], [15] or treatment regimens [16], [17]. In order to understand the effect of high dose cyclophosphamide on different genes, we employed the DNA microarray to investigate the gene expression profile in peripheral mononuclear cells of patients suffering from haematological malignancies and undergoing conditioning regimen consisting of Cy followed by TBI. We followed the gene profile pre-, during and after treatment.

## Materials and Methods

### Patients and Treatment

Eleven patients were enrolled in this study. The patients were admitted at the Centre for Allogeneic Stem Cell Transplantation (CAST), Karolinska University Hospital-Huddinge. The study was approved by the ethical committee of Karolinska Institutet (616/03) and written informed consent was obtained from the patients or, in case of paediatric patients, their parents. Twelve samples from healthy donors, who had given their written consent, were run concomitantly as negative controls. The ethics committee approved the consent procedure. Six of these patients were diagnosed with acute lymphocytic leukaemia (ALL), two with acute myeloid leukaemia (AML), two with T-cell lymphoma and one with chronic lymphocytic leukaemia (CLL). Patient characteristics are presented in Table 1.

**Table 1:** Patient characteristics.

Patient Code	Age (years)	Diagnosis	Conditioning regimen	Stem cells source	Donor	CD 34 dose/Kg	Disease status at HSCT	Acute GVHD	Outcome	Cause of death
**P 1**	57	B-CLL	CP+fTBI (6 Gy)+ Alemtuzumab	PBSC	Sib	14.7×10^6^	Transformed	Grade II	† 10 months	Relapse
**P 2**	38	T cell lymphoma	CP+fTBI	BM	Sib	2.9×10^8^	PR	Grade II	† 19 months	Relapse
**P 3**	31	AML	CP+fTBI+ATG	BM	MUD	2×10^6^	CR2	Grade II	† 6 months	Pneumonia
**P 4**	10	T-ALL	CP+fTBI+ATG	BM	Sib	6.48×10^8^	CR1	Grade I	† 12 months	Relapse
**P 5**	26	Pre B-All	CP+fTBI+ATG	PBSC	MUD	13.5×10^6^	CR2	Grade II	† 35 months	Relapse
**P 6**	19	ALL	CP+fTBI+ATG	PBSC	MUD	13.5×10^6^	CR3	No	† 9 months	Relapse & pneumonia
**P 7**	51	AML	CP+fTBI+ATG	PBSC	Sib	10.6×10^6^	Refractory	Grade I	Alive 7.5 years	–
**P 8**	25	Pre B-ALL	CP+fTBI+ATG	PBSC	Sib	7.3×10^6^	CR2	Grade II	Alive 7.5 years	–
**P 9**	14	T-ALL	CP+fTBI+ATG	PBSC	MUD	19.9×10^6^	CR2	Grade II	Alive 7.2 years	–
**P 10**	41	T cell lymphoma	CP+fTBI+ATG	PBSC	MUD	9.3×10^6^	Relapse	Grade I	† 51 days	Invasive fungal infection
**P 11**	26	T-ALL	CP+fTBI+ATG	DUCB	DUCB	0.5×10^5^0.2×10^5^	CR2	Grade I	† 11 months	Relapse

**Abbreviations:** GVHD, graft versus host disease; P, patient; CLL, chronic lymphoblastic leukaemia; AML, acute myeloid leukaemia; ALL, acute lymphoblastic leukaemia; CP, cyclophosphamide; fTBI, fractionated total body irradiation; ATG, antithymocyte globulin; PBSC, peripheral blood stem cells; BM, bone marrow; Sib, HLA-identical sibling; MUD, matched unrelated donor; DUCB, Double umbilical cord blood; CR, complete remission; PR, partial remission;

† survival.

All the patients received an i.v. infusion of Cy 60 mg/kg/day once daily for two days followed by fractionated TBI 3 Gy (gray) x 4, except the first patient who only received 6 Gy in total. Blood samples were collected from each patient before the start of Cy infusion, 6 h after the first dose of Cy, before and 6 h after the second dose of Cy. Samples have been numbered with the patient’s number followed by −1, −2, −3 and −4.

All patients with unrelated donors received antithymocyte globulin (ATG, Thymoglobulin, Genzyme, Cambridge, MA, USA), at a total dose of 6 mg/kg given at day −4–−1 during the conditioning treatment. The single exception was the first patient, who had a sibling donor and received Alemtuzumab 30 mg × 1 due to CD52 expression of the leukaemia.

### GVHD Prophylaxis

GVHD prophylaxis consisted of cyclosporine (CsA) in combination with four doses of methotrexate (MTX) [18]. During the first month, blood CsA levels were kept at 100 ng/mL when a sibling donor was used, and at 200–300 ng/mL when an unrelated donor was used. In the absence of GVHD, CsA was discontinued after three to four months for patients with sibling donors and six months for patients with MUD.

### Diagnosis and Treatment of GVHD

Acute and chronic GVHD were diagnosed on the basis of clinical symptoms and/or biopsies (skin, liver, gastrointestinal tract, or oral mucosa) according to standard criteria [19]. The patients were treated for grade I acute GVHD with prednisolone, starting at a dosage of 2 mg/kg/day, which was successively lowered after the initial response. Chronic GVHD was initially treated with CsA and steroids. In most cases, daily prednisone at 1 mg/kg per day and daily CsA at 10 mg/kg per day were used [20].

### Blood Sampling and RNA Extraction

Blood samples for RNA extraction were collected in PAXgene tubes (BD, Stockholm, Sweden). RNA was extracted using QuickPrep Total RNA Extraction Kit (GE Life Sciences, Uppsala, Sweden) according to the manufacturer’s instructions and then quantified by measuring the absorbance at 260 and 280 nm. All samples were stored at −180°C.

### Gene Expression Assay and Analysis

Purified mRNA was subjected to analysis of global gene expression by using NimbleGen microarrays (Roche Diagnostics Scandinavia, Bromma, Sweden). Data were analyzed using GeneSpring GX (Agilent, CA, USA). The expression data were normalized using quantile normalization and the gene expression data were generated using the Robust Multichip Average algorithm. Significant differences in gene expression were determined by ANOVA. The selection threshold of a false discovery rate (FDR) was <5% and the fold change in the SAM output result was >2. The complete data (ID no. 20051907) is available via the GEO database with the accession number “GSE51907”.

Pathway identification and reporting was performed using IPA software (Ingenuity, Qiagen, CA, USA) and Kyoto Encyclopaedia of Genes and Genomes software (KEGG) (Kyoto University Bioinformatics Centre, Japan).

### cDNA Synthesis and Real Time PCR (qRTPCR)

Two micrograms of RNA were reversed transcribed to complementary DNA (cDNA) using the TaqMan Reverse Transcriptase cDNA Kit (Applied Biosystems, Roche, NJ, USA) and then stored at −80°C. TaqMan gene expression assay (Applied Biosystems, Stockholm, Sweden) was performed by means of the FAM dye labelling system according to the manufacturer’s instructions. The assay was performed for the selected genes that showed significantly high expression, ANGPTL1 and c-JUN (assay IDs; Hs00559786_m1 and Hs01103582_s1, respectively) and GAPDH (assay ID; Hs02758991_g1) as a housekeeping gene (Applied Biosystems, Stockholm, Sweden). Real time PCR (qRTPCR) reactions were performed in a 384-well plate thermal cycler ABI 7900 (Applied Biosystems, Stockholm, Sweden) in a total volume of 10 µL and results were normalized against the housekeeping gene GAPDH. Twelve samples from healthy donors were run concomitantly as negative controls.

## Results

### Outcome of HSCT after Conditioning with Cy and TBI

As shown in Table 1, all patients received allogeneic HSCT either from their siblings (5 patients) or from MHC-matched unrelated donors (MUD) (5 patients) after conditioning with Cy and TBI. Stem cells from peripheral blood (PBSC) were given to 7 patients, and 3 patients received bone marrow (BM). One patient received a double umbilical cord blood (DUCB) transfusion.

Most of the patients were in remission at the time of transplantation (8/11, 72.7%). Acute GVHD, either grade I or grade II, was reported in 10 patients (90.9%). The overall survival was 27.3% (3/11 patients) and relapse was the main cause of death in most of the patients (6/8, 75%).

### Identification of Differentially Expressed Genes and Gene Clusters Related to Cy Treatment

**Figure 1:**
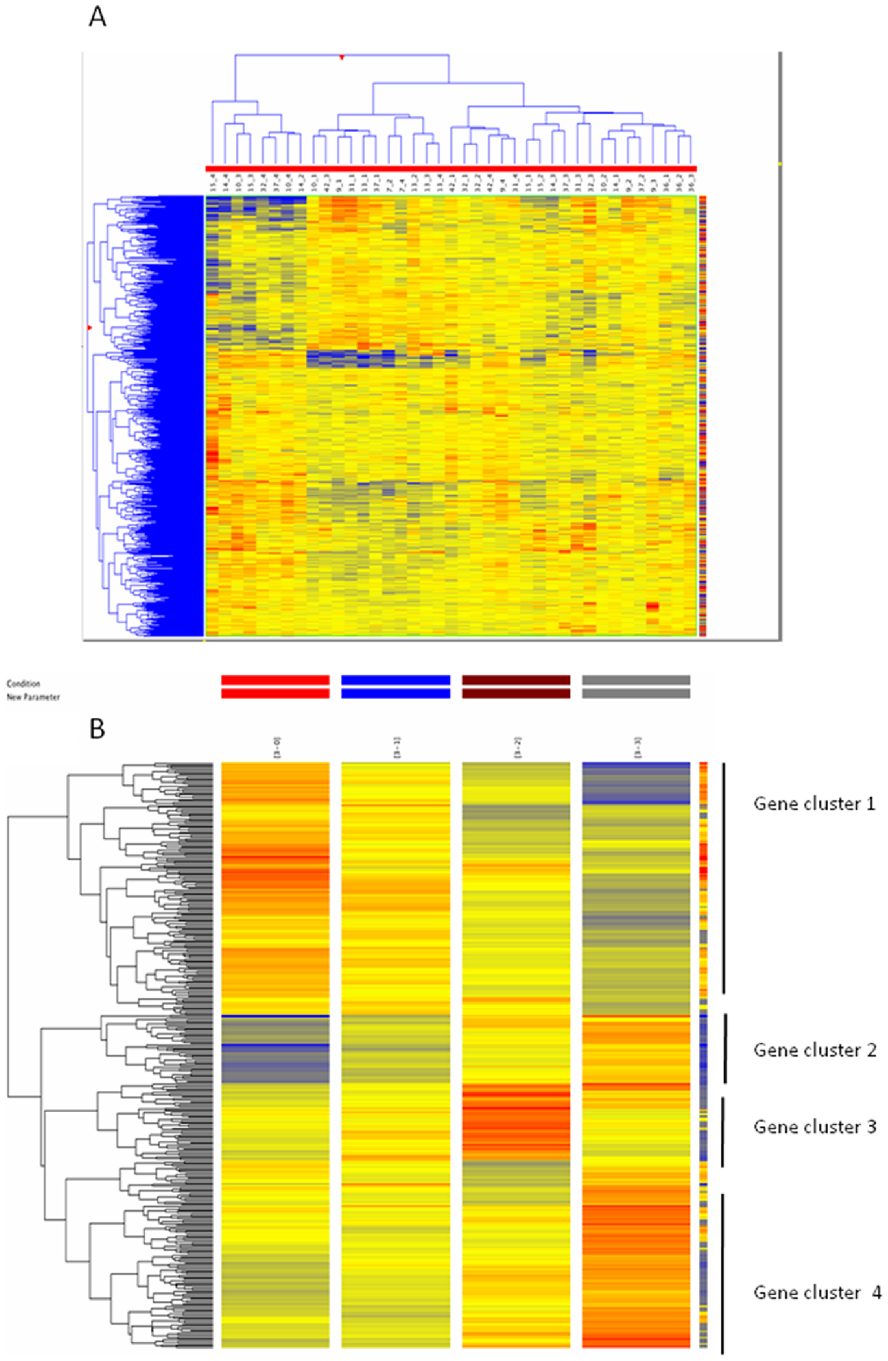
Heat map of patient gene expression during Cy treatment. Genes expression for genes specific for Cy before and though the treatment (A). Differential expression of a group of 299 genes was identified as being specific for Cy treatment. After subjecting these genes to hierarchical clustering analysis, 4 clusters of up- and down-regulated genes were identified as: highly down-regulated genes (cluster 1), highly up-regulated genes (cluster 2), early up-regulated but later normalized genes (cluster 3) and moderately up-regulated genes (cluster 4) as seen in B.

We assessed the overall patterns of gene expression in 11 patients with haematological malignancies treated with Cy. The assessment was performed utilizing a hierarchical clustering analysis of the signal ratios of all arrays. The heat map representing array clustering based on normalized probe intensity showed a high inter-individual variation, as expected (Figure 1A). In the present study we observed variation in several thousands of genes during and after Cy treatment. However, after fold-change filtering (at least 2-fold compared to time 0, i.e. before treatment), differential expression of a group of 299 genes was identified as being specific for Cy treatment. By subjecting these genes to hierarchical clustering analysis, we were able to identify 4 clusters of up- and down-regulated genes which matched the chronological cascade of gene expression by cyclophosphamide treatment: highly down-regulated genes (cluster 1), highly up-regulated genes (cluster 2), early up-regulated but later normalized genes (cluster 3) and moderately up-regulated genes (cluster 4) (Figure 1B).

#### Highly down-regulated genes (cluster 1)

**Figure 2:**
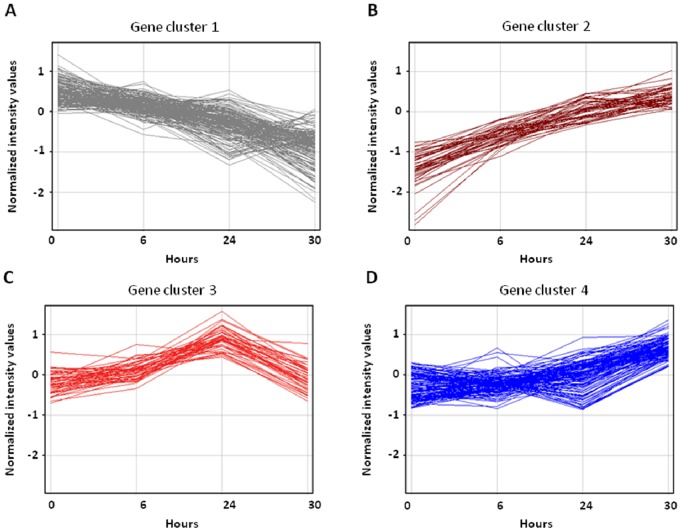
Gene clusters in relation to Cy treatment. The expression of Cy treatment specific genes at 6(30 h) was normalized to the pre-treatment and divided to the following clusters: Cluster 1 showed highly down-regulated genes throughout the treatment (A). Cluster 2 showed highly up-regulated genes throughout the treatment (B). Cluster 3 showed early up-regulated but later normalized genes (C). Cluster 4 showed moderately up-regulated genes (D).

**Figure 3:**
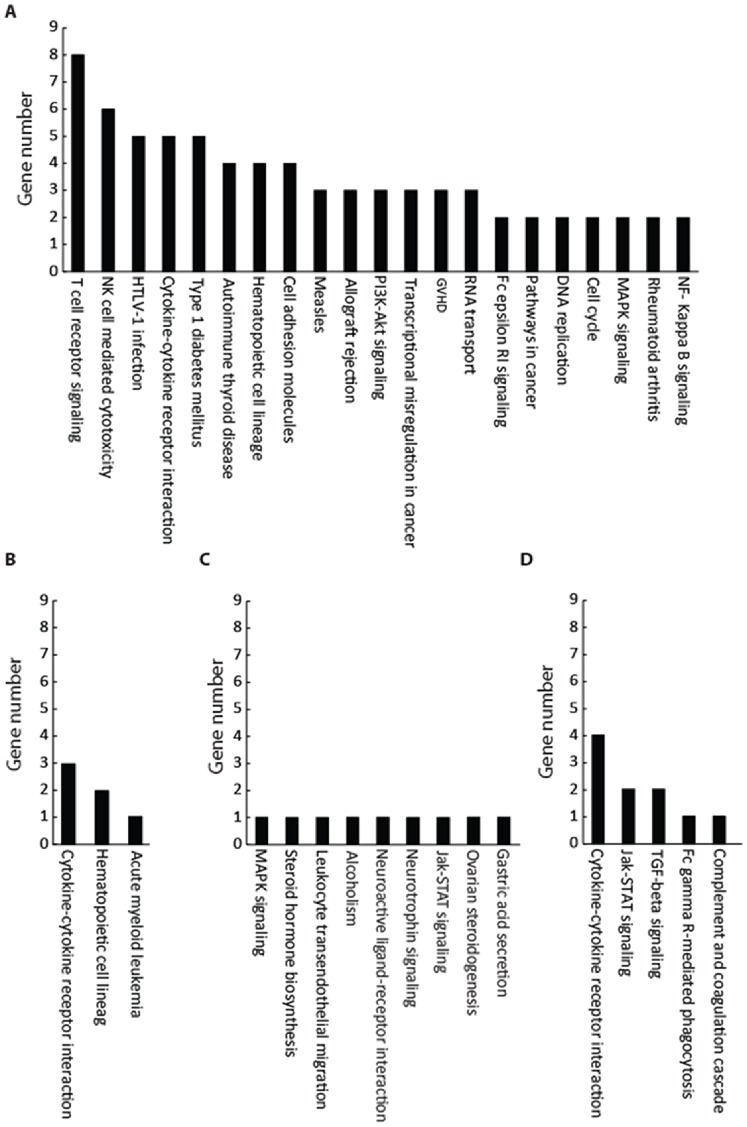
The pathways related to each cluster and number of genes involved in each cluster. Cluster 1 for highly down-regulated genes throughout the treatment (A) included the highest number of genes. The majority of these genes belonged to the immune system and its functions. Cluster 2 for highly up-regulated genes throughout the treatment (B), the majority of these genes are involved in 3 important biological pathways involving cytokine-cytokine receptor interaction, transcriptional misregulation in cancer and hematopoietic cell lineage. Cluster 3 showed early up-regulated but later normalized genes (C), these genes were more related to biological pathways including Jak-STAT and MAPK signalling. Cluster 4 showed moderately up-regulated genes (D), the pathway analysis demonstrated that several pathways including cytokine-cytokine receptor interaction, Jak-STAT signalling pathway and TGF-beta signalling are involved.

The first cluster represents genes that were relatively up-regulated in all patients prior to cyclophosphamide treatment; however, this cluster was down-regulated at 6 h post administration followed by a pronounced decrease in expression at 30 h (i.e. 6 h after the second dose; Fig. 2A). This cluster possessed the highest number of genes (139 genes, Table 2). The majority of these genes belonged to the immune system and its functions (Table 3). Moreover, further analysis of biological pathways related to these genes showed that a majority of immune- (e.g. T cell receptor signalling, natural killer cell mediated cytotoxicity and graft rejection), autoimmune- (e.g. autoimmune thyroid disease pathway, type 1 diabetes mellitus and rheumatoid arthritis) and inflammation- (e.g., GVHD and NF-kB signalling) related processes were down-regulated by the Cy treatment (Figure 3A).

**Table 2:** All genes specific for CP treatment arranged in 4 clusters.

Cluster 1						Cluster 2		Cluster 3		Cluster 4			
TNIK	DYRK2	TMEM116	IL5RA	PTPN4	MRAS	RASGEF1A	FKBP5	SEC14L2	ST8SIA2	TRIO	MCEMP1	AP3B2	ANGPTL7
HEG1	ST6GALNAC6	ITGA4	CD3D	RORA	TKTL1	FKBP5	FLT3	PSORS1C2	ST8SIA5	HFE	MYO10	KL	ZNF708
STK39	HLA-DQA1	TNIK	CD3E	XCL1	GNLY	VSIG4	CD163	KLK13	OR12D2	ZNF396	USP37	HIST1H1T	ZNF500
RALGDS	MCM7	PTPLAD1	IL2RB	XCL2	GPR171	CRISPLD2	ASPH	CHST1	NR2F2	SEC24D	HYLS1	H3F3B	SAMSN1
AES	IL32	XPO5	ITGA4	UNG	OLFM1	HIST1H2BC	IL1R2	WFDC1	KCNK10	RNF175	SLC3A1	ABCA1	ITPKC
CD244	MAGEH1	SEPT11	GATA3	RUVBL1	KIR2DS1	ECHDC3	PFKFB2	ADRA2A	WFDC1	FLJ36031	BRCA2	MCAM	MGC11332
GSDML	SPN	MASA	ABLIM1	PEX11A	TNIK	GRB10	VSIG4	DYRK3	SPRY4	CCDC62	F8	HS2ST1	ZNF558
KLRC3	LCK	KIR2DL1	TARP	DOK2	NUP205	APBB2	ADAMTS2	THY1	FLJ39155	TREX2	LPL	SOCS5	DNAJB7
GSDML	FCER1A	XCL2	AKAP2	MFHAS1	POLR1A	CD177	DAAM2	FBN1	CPLX4	FLJ46419	HPGD	SYCP2	ZNF396
POLR1B	NKG7	CTLA4	SSBP3	DDX1	ZNFN1A2	BCAT1	THEDC1	SPATS1	NNAT	EDA2R	FLJ44796	ITGB3BP	ZNF718
PCNT	PRKACB	XCL1	IL32	PRF1	KLF12	KCNE1	ASPH	OR10J1	PTHLH	FLJ21963	ZNF613	CPEB3	MCEMP1
MRPL1	CD96	CCR4	LAT	CSF1R	CD244	PFKFB2	CD177	GP2	ZCCHC13	IL13RA1	CNGA4	KIF1B	MFSD4
PRKACB	SH2D1B	PRSS21	SAMD3	GPR15	ABI3	IL18R1	ADAMTS2	TRPV4	DUB3	ITGB3BP	ARL1	PPARG	TMEM119
FLJ20701	GNLY	GPR162	CTSW	LCK	TEX10	KCNE1	RASGEF1A	TBX5	HSD17B2	ARHGAP29	BMPR2	UBE2J1	CCDC62
ABLIM1	CDCA7	IGHG1	CX3CR1	MAF	FLJ20701	CA4	APBB2	OR56A1	DMP1	KLHL8	KLF9	CNR1	FLJ43806
GZMB	ZNFN1A2	KLRC2	CD38	CCR4	HEATR1	GRB10	MGC34646	NTRK2	MALL	DC36	FOXC1	IL1RL1	FLJ44313
ZNFN1A2	MRPL1	CD28	FKBP4	SMAD7	GSDML	THEDC1	CD163	DRP2		RPS27L	INHBB	PRPF39	TMED8
RASGRP1	CBLB	CYSLTR2	GZMK	MCM4	EEFSEC	ADM	ASPH			CRISPLD2	KCNMA1	FEM1C	HPGD
PPIE	KCNA3	MYBL1	HDC	GZMA	DDX31	ALOX15B	IL1R2			SLC10A1	TACSTD1	ACVR1B	STRA13
CETN3	MLLT3	LPAL2	NID1	GNLY	LYNX1	CA12	IL18R1			CNR1	TIMP4	SRGAP1	HPGD
NUP37	MTAC2D1	NFATC2	PRKACB	MLC1	PIK3C2B	TPST1				FEZ2	IL1RL1	LRRN1	FLJ42562
NCALD	INADL	IL5RA	LCK	MYBL1	KIR2DS1					TMEM119	ACSL3	GCN5L2	CNGA4
TMEM116	CBLB	FLJ46020								SYCP2	FMN1		

**Table 3:** Pathways reported in each cluster and genes involved in each of them.

Cluster	Pathways	Genes
**Cluster 1**	T cell receptor signalling	CTLA4, CD3ε, CD3δ, CD28, Lck, LAT, RasGRP1, NFAT
	Natural killer cell mediated cytotoxicity	Lck, LAT, NFAT, 2B4, GZMB, PRF1
	HTLV-1 infection	CD3, IL2R, NFAT, Ras, Lck
	Cytokine-cytokine receptor interaction	CX3CR1, CCR4, IL5RA, IL2RB, CSF1R
	Type 1 diabetes mellitus	INS, MHC-II, CD28, PRF1, GZMB
	Autoimmune thyroid disease	CTLA4, CD28, PRF1, GZMB
	Hematopoietic cell lineage	CD3, CD115, IL5RA, CD49
	Cell adhesion molecules	CD28, CTLA4, SPN, ITGA4
	Measles	CD3, CD28, IL2R
	Allograft rejection	CD28, PRF1, GZMB
	PI3K-Akt signalling	RTK, Cytokine R, ITG A
	Transcriptional misregulation in cancer	LMO2, PAX3, PAX7
	Graft-versus-host disease	CD28, PRF1, GZMB
	RNA transport	Exp5, Nup37, Nup205
	Fc epsilon RI signalling	FcεRIα, LAT
	Pathways in cancer	Ra1GDS, MCSFR
	DNA replication	Mcm4, Mcm7
	Cell cycle	Mcm4, Mcm7
	MAPK signalling	RasGRP1, Ras
	Rheumatoid arthritis	CD28, CTLA4
	NF- Kappa B signalling	Lck, LAT
**Cluster 2**	Cytokine-cytokine receptor interaction	FLT3, IL1R2, IL18R1
	Hematopoietic cell lineage	CD135, CD121
	Acute myeloid leukaemia	AML1
**Cluster 3**	MAPK signalling	TrkA/B
	Steroid hormone biosynthesis	17beta-estradiol
	Gastric acid secretion	KCN
	Alcoholism	TrkB
	Leukocyte transendothelial migration	Thyl
	Neuroactive ligand-receptor interaction	ADR
	Neurotrophin signalling	TrkB
	Jak-STAT signalling	Sprouty
	Ovarian steroidogenesis	17β-HSD
	MAPK signalling	TrkA/B
**Cluster 4**	Cytokine-cytokine receptor interaction	IL13RA1, XEDAR, ACVR1B, BMPR2
	Jak-STAT signalling	CytokineR, SOCS
	TGF-beta signalling	BMPRII, ActivinR1
	Fc gamma R-mediated phagocytosis	Myosin X
	Complement and coagulation cascade	coagulation factor VIII

#### Highly up-regulated genes (cluster 2)

In contrast to cluster 1, this group of genes exhibited a constant and high up-regulation in response to Cy treatment (Fig. 2B). In this cluster, 41 up-regulated genes were identified (Table 2). The majority of these genes are involved in 3 important biological pathways involving cytokine-cytokine receptor interaction, transcriptional misregulation in cancer and hematopoietic cell lineage (Table 3 and Fig. 3B). Only one gene in this cluster was found to be related to the acute myeloid leukaemia pathway (Table 3 and Fig. 3B).

#### Early up-regulated but later normalized genes (cluster 3)

This group of genes exhibited significant up-regulation at an early time point in Cy treatment, but the expression was later normalized to the same level as before the start of treatment (Fig. 2C). This cluster included 33 genes (Table 2). Analysis of biological pathways related to these genes showed that although several pathways are involved (Table 3), only one gene in each pathway is affected by treatment with Cy (Fig. 3C).

#### Moderately up-regulated genes (cluster 4)

Finally, treatment with Cy resulted in moderate up-regulation of a group of genes, mainly by the end of treatment (6 hr after the second dose; Fig. 2D). There were 90 genes in this cluster (Table 2) and the biological pathway analysis demonstrated that several pathways including cytokine-cytokine receptor interaction (4 genes), Jak-STAT signalling pathway (2 genes) and TGF-beta signalling pathway (2 genes) are related to this cluster (Table 3 and Fig. 3D).

### Disease-related Common Up-regulated Genes

**Figure 4:**
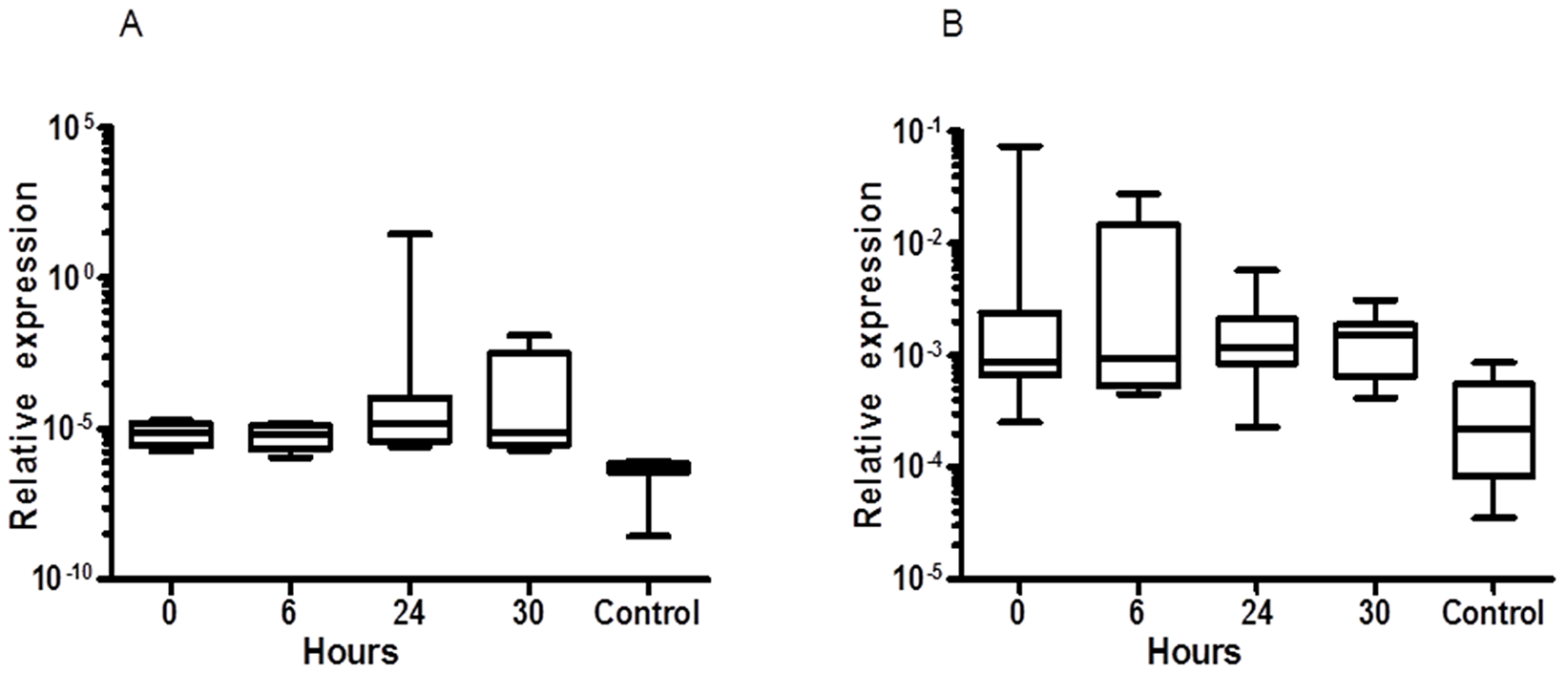
Effect of cyclophosphamide treatment on ANGPTL1 and c-JUN. The relative expression as measured by qRTPCR (normalized to GAPDH) of disease-related up-regulated genes, ANGPTL1 (A) and c-JUN (B), compared to normal subjects. Cy treatment did not affect the up-regulation of ANGPTL1 and c-JUN.

Hierarchical clustering analysis showed that 2 genes, angiopoietin-like-1 (ANGPTL1) and c-JUN proto-oncogene (c-JUN), were considerably up-regulated prior to, during and after treatment with Cy in all tested patients. Further, these results were confirmed by qRTPCR and showed that these two genes were up-regulated in the patients treated with Cy. Figure 4A shows the results obtained from qRTPCR analysis of ANGPTL1, the gene expression was up-regulated from time 0 e.g. before Cy treatment and continued to be at a high expression level on 6 h after the first dose, 24 h (time before second dose) and at 6 h after the second dose (30 h) compared to control (healthy subjects). As ANGPTL1, and independent of Cy treatment, c-JUN gene expression was also found to be high expressed compared to healthy subjects (Fig. 4B).

## Discussion

This is the first global gene expression profiling study of patients suffering from haematological malignancies, treated with Cy as a conditioning regimen and undergoing HSCT. The data analysis has generated comprehensive knowledge that can be employed in understanding the rationales by which Cy is used as an immunosuppressive/immunoregulatory or conditioning agent.

Our first set of findings that treatment with Cy down-regulated the expression of several genes mapped to immune/autoimmune activation, allograft rejection and GVHD strongly confirm that this alkylating agent is a potent immunosuppressive agent. In this connection, the most noticeable down-regulated genes are CD3, CD28, CTLA4, MHC II, PRF1, GZMB and IL-2R.

CD3 molecule is a complex protein that is expressed as a co-receptor in all mature T lymphocytes and is a subset of NK cells [21]. This co-receptor molecule plays a key role in T cell activation and is therefore a potential target for several drugs, including monoclonal antibodies for the treatment of different autoimmune diseases [22], [23]. In this respect, down-regulation of CD3 gene expression implies that the initial event of T cell activation, which requires the formation of a complex consisting of CD3 and T cell receptor, is impaired upon treatment with Cy.

CD28 and CTLA4 are two surface molecules that play a crucial role in activation and subsequent regulation of cell-mediated immune responses [24]. CD28 is constitutively expressed on the surface of T cells and provides a key co-stimulatory signal upon interaction with CD80 (B7-1) and CD86 (B7-2) on antigen-presenting cells [25]. In contrast, CTLA4 is transiently expressed in activated T cells. CTLA4, by binding to CD80 or CD86, delivers negative signals, which leads to T cell inactivation [25]. Our observation that treatment with Cy down-regulated the expression of both CD28 and CTLA4 suggests that this drug exerts dual effects on T cells as it suppresses the early phase of T cell activation as well as prolongs the activity of effector T cells.

MHC II molecules (major histocompatibility complex class II molecules) are the key molecules involved in presenting antigens to CD4+ T cells. These molecules are constitutively expressed in professional (macrophages, dendritic and B cells) and non-professional (thymic epithelial cells) antigen presenting cells [26]. By binding to foreign peptides, these molecules provide “signal 1” for activation of CD4+ T cells. Thus, down-regulation of the expression of MHC II in Cy treated patients implies that this drug prevents T cell activation by impairing the process of MHC II mediated antigen presentation. Furthermore, this mechanism of action can also explain the efficacy of Cy in the treatment of autoimmune diseases (e.g. RA and SLE) where antigen presentation and antigen presenting cells (in particular B-cells) play a crucial role.

Due to its effect on autoimmune diseases, cyclophosphamide has recently been used in high doses after HSCT to prevent graft rejection and GVHD [27]. Moreover, HLA matching does not seem to be important if the patient receives post-transplantation Cy, which is a great advantage to patients lacking conventional donors [28], [29].

The PRF1 (perforin-1) gene encodes a cytolytic protein, which is found in cytotoxic T cells and NK cells. PRF1 shares similarities in both structure and function with complement component 9 (C9) [30]. Like BRF1, GZMB (granzyme B) is a protease expressed by cytotoxic T lymphocytes and NK cells and induces apoptosis on target cells [31]. It has been demonstrated that granzyme can access its target cells through pores formed by perforin [32]. Our observation that the expression of BRF1 and GZMB genes are down-regulated upon treatment with Cy strongly suggests that cytotoxic activity of the immune cells mainly mediated by CD8+ T and NK cells is also lessened by Cy.

IL-2R (Interleukin-2 receptor) is expressed on the activated T cells as well as regulatory T (T_reg_) cells (also known as suppressor T cells). Upon binding to IL-2, IL-2R promotes cell cycle progression through phase G1 of the cell cycle, which leads to the onset of DNA synthesis and replication [33]. Therefore, down-regulation of IL-2R gene expression in Cy treated patients may prevent alloreactivity against donor hematopoietic stem cells. Furthermore, this reduction in IL-2R expression might also attenuate the number of T_reg_ cells, which are known to play an unfavourable role in malignancies [34], [35]. In line with this statement, it has been shown that Cy can suppress T_reg_ cells and allow more effective induction of antitumor immune responses [36].

In addition to the genes related to the immune system, our findings also demonstrated that treatment with Cy down-regulates the expression of several genes (e.g., Ras, LMO2, MCM4 and MCM7) that are related to cancer development and cell cycle progression. For instance, Ras (rat sarcoma) oncoproteins are known to be responsible for signal transmission inside the cells and for participating in cell growth, differentiation and survival [37]. Oncogenic mutations in Ras genes have been detected in several human cancers [38], [39].

The LMO2 (LIM domain only 2) gene encodes a cysteine-rich, two protein structural domain that plays an important role in hematopoietic development; moreover, its ectopic expression in T cells leads to the onset of acute lymphoblastic leukaemia (ALL) [40]. In mice, LMO2 induced precancerous stem cells and even initiated leukaemia (T-ALL) by inducing thymocyte self-renewal [41], [42].

Finally, minichromosome maintenance proteins (MCM) 4 and 7 are known to be essential for the initiation of genomic replication [43] and their down-regulation during Cy treatment confirms the ability of this drug to reduce cancer size by slowing cell replication. MCM4 and MCM7 were found in both DNA replication and cell cycle pathways. Thus, Cy induced down regulation in Ras, LMO2, MCM4 and MCM7 genes might shed light on the mechanisms underlying the anti-cancer effects of Cy.

Our findings in this study have also demonstrated that in addition to the down-regulated genes, several genes are up-regulated during treatment with Cy. Most of these genes are immune-related receptor genes, e.g. IL1R2 (interleukin 1 receptor, type II or CD121b), IL18R1 (interleukin-18 receptor 1 or CDw218a) and FLT3 (Fms-like tyrosine kinase 3, or CD135). IL1R2 is a protein expressed on B cells, monocytes and neutrophils and functions as a molecular decoy that sequesters IL-1β and blocks the initiation of downstream signalling, thereby preventing inflammation [44]. Moschella *et al*. have reported that IL-1β was increased to reach maximum concentration at day 3 after Cy administration which is in good agreement with our finding [45]. On the other hand, IL18R1 is a cytokine receptor that specifically binds interleukin 18 (IL18) and is essential for IL18 mediated signal transduction. IFN-α as well as IL12 are reported to induce the expression of this receptor in NK and T cells. Interestingly, IL18R1 and IL1R2 genes along with three other members of the interleukin 1 receptor family, including IL1R1, ILRL2 (IL-1Rrp2), and IL1RL1 (T1/ST2) form a gene cluster on chromosome 2q [46]. Thus, Cy induced increase in expression of IL1R2 and IL18R1 suggests that cytokine receptor genes located on chromosome 2q are susceptible to Cy. In a recent publication, Moscella *et al.* have reported that Cy has activated IFN-α signature and IFN-α –induced proinflammatory mediators. Moreover, Cy also has induced expansion and activation of IL18R1 and other receptors [47]. These results confirm our finding; however, further studies are required to confirm mechanisms underlying this hypothesis.

FLT3 is a protein expressed on the surface of many hematopoietic progenitor cells and plays an important role in the development of B and T progenitor cells [48]. However, it remains to be elucidated if increased expression of FLT3 implies that treatment with Cy might lead either directly or indirectly to mobilization of hematopoietic progenitor cells to the periphery.

Our results showed a significant increase (confirmed by qRTPCR) in the expression of angiopoietin-related protein 1 (ANGPTL1) and c-JUN proto-oncogene (c-JUN) genes in all patients. The high expression was independent of Cy treatment. These two genes are known to play an important role in cancer, i.e. ANGPTL1, which is a member of the vascular endothelial growth factor family, was reported to mediate a defence mechanism against cancer growth and metastasis. In this respect, Kuo *et al.* reported the inverse correlation between the expression of ANGPTL1 and cancer invasion and lymph node metastasis in lung cancer patients and experimental cancer models [49].

Overexpression of c-JUN has been shown in several human cancer types such as non-small cell lung cancer, breast cancer, colon cancer and lymphomas [50]–[53]. Moreover, c-JUN was reported to be associated with proliferation and angiogenesis in invasive breast cancer [54]. Jiao et al. have reported that c-JUN induced epithelial cellular invasion in breast cancer [55]. Cancer cells are rapidly dividing and c-JUN is important for progression through the G1 phase of the cell cycle [56]. c-JUN antagonizes P53 expression which is a cell cycle arrest inducer [57]. Moreover, c-JUN is an apoptosis down-regulator, which is important for cancer cell survival [56]. c-JUN was reported to promotes BCR-ABL induced lymphoid leukemia [58]. Furthermore, the expression of c-JUN was reported to be enhanced in chemotherapy resistant tumors [59]–[61].

In the present investigation, the high expression of ANGPTL1 and c-JUN genes was observed throughout the treatment with no effect of Cy therapy on these genes. Thus, based on our findings and the reported studies, we propose that these genes might be considered as potential markers for therapeutic efficacy connected to haematological malignancies. In addition, we strongly believe that targeting the gene expression of c-JUN might have therapeutic potential for these diseases [62], [63].

In conclusion, our results in the present study provide significant information about the alterations in gene expression caused by Cy treatment. We demonstrate here that Cy induces both down- and up-regulation in genes, mainly belonging to the immune system. This knowledge can be expanded further to evaluate which Cy metabolites are responsible for these effects and how Cy can be employed to target specific immune function in order to optimize Cy treatment and/or to minimize the treatment related toxicity and hence enhance HSCT clinical outcome. Our findings also show Cy independent over expression of some genes that have been reported in solid tumours. These may suggest that these genes may be used as a target treatment for haematological malignancies. However, several studies are warranted.

## References

[1] Burt RK, Loh Y, Pearce W, Beohar N, Barr WG, et al (2008) Clinical applications of blood-derived and marrow-derived stem cells for nonmalignant diseases. JAMA : the journal of the American Medical Association 299: 925–936. 18314435 10.1001/jama.299.8.925

[2] Reynolds M, McCann SR (1989) A comparison between regimens containing chemotherapy alone (busulfan and cyclophosphamide) and chemotherapy (V. RAPID) plus total body irradiation on marrow engraftment following allogeneic bone marrow transplantation. Eur J Haematol 43: 314–320. 2684682 10.1111/j.1600-0609.1989.tb00305.x

[3] Daleboudt GM, Reinders ME, den Hartigh J, Huizinga TW, Rabelink AJ, et al (2013) Concentration-controlled treatment of lupus nephritis with mycophenolate mofetil. Lupus 22: 171–179. 23257398 10.1177/0961203312469261

[4] Binotto G, Trentin L, Semenzato G (2003) Ifosfamide and cyclophosphamide: effects on immunosurveillance. Oncology 65: 17–20. 14586142 10.1159/000073353

[5] Sladek NE (1988) Metabolism of oxazaphosphorines. Pharmacology & therapeutics 37: 301–355. 3290910 10.1016/0163-7258(88)90004-6

[6] Cho JY, Lim HS, Chung JY, Yu KS, Kim JR, et al (2004) Haplotype structure and allele frequencies of CYP2B6 in a Korean population. Drug Metabolism and Disposition 32: 1341–1344. 15383491 10.1124/dmd.104.001107

[7] Chang TK, Weber GF, Crespi CL, Waxman DJ (1993) Differential activation of cyclophosphamide and ifosphamide by cytochromes P-450 2B and 3A in human liver microsomes. Cancer Res 53: 5629–5637. 8242617

[8] Ren S, Yang JS, Kalhorn TF, Slattery JT (1997) Oxidation of cyclophosphamide to 4-hydroxycyclophosphamide and deschloroethylcyclophosphamide in human liver microsomes. Cancer Res 57: 4229–4235. 9331082

[9] Xie H, Griskevicius L, Stahle L, Hassan Z, Yasar U, et al (2006) Pharmacogenetics of cyclophosphamide in patients with hematological malignancies. Eur J Pharm Sci 27: 54–61. 16183265 10.1016/j.ejps.2005.08.008

[10] Klein K, Lang T, Saussele T, Barbosa-Sicard E, Schunck WH, et al (2005) Genetic variability of CYP2B6 in populations of African and Asian origin: allele frequencies, novel functional variants, and possible implications for anti-HIV therapy with efavirenz. Pharmacogenet Genomics 15: 861–873. 16272958 10.1097/01213011-200512000-00004

[11] Rotger M, Colombo S, Furrer H, Bleiber G, Buclin T, et al (2005) Influence of CYP2B6 polymorphism on plasma and intracellular concentrations and toxicity of efavirenz and nevirapine in HIV-infected patients. Pharmacogenetics and genomics 15: 1–5. 15864119 10.1097/01213011-200501000-00001

[12] Tsuchiya K, Gatanaga H, Tachikawa N, Teruya K, Kikuchi Y, et al (2004) Homozygous CYP2B6 *6 (Q172H and K262R) correlates with high plasma efavirenz concentrations in HIV-1 patients treated with standard efavirenz-containing regimens. Biochem Biophys Res Commun 319: 1322–1326. 15194512 10.1016/j.bbrc.2004.05.116

[13] Eroglu C, Pala C, Kaynar L, Yaray K, Aksozen MT, et al. (2013) Comparison of total body irradiation plus cyclophosphamide with busulfan plus cyclophosphamide as conditioning regimens in patients with acute lymphoblastic leukemia undergoing allogeneic hematopoietic stem cell transplant. Leuk Lymphoma. 10.3109/10428194.2013.779691 23442062

[14] de Jonge HJ, Huls G, de Bont ES (2011) Gene expression profiling in acute myeloid leukaemia. The Netherlands journal of medicine 69: 167–176. 21527803

[15] Mills K (2008) Gene expression profiling for the diagnosis and prognosis of acute myeloid leukaemia. Front Biosci 13: 4605–4616. 18508532 10.2741/3026

[16] Khokher S, Qureshi MU, Mahmood S, Nagi AH (2013) Association of immunohistochemically defined molecular subtypes with clinical response to presurgical chemotherapy in patients with advanced breast cancer. Asian Pac J Cancer Prev 14: 3223–3228. 23803108 10.7314/apjcp.2013.14.5.3223

[17] Rimsza LM, Leblanc ML, Unger JM, Miller TP, Grogan TM, et al (2008) Gene expression predicts overall survival in paraffin-embedded tissues of diffuse large B-cell lymphoma treated with R-CHOP. Blood 112: 3425–3433. 18544678 10.1182/blood-2008-02-137372 PMC4467875

[18] Ringden O, Horowitz MM, Sondel P, Gale RP, Biggs JC, et al (1993) Methotrexate, cyclosporine, or both to prevent graft-versus-host disease after HLA-identical sibling bone marrow transplants for early leukemia? Blood 81: 1094–1101. 8427991

[19] Przepiorka D, Weisdorf D, Martin P, Klingemann HG, Beatty P, et al (1995) 1994 Consensus Conference on Acute GVHD Grading. Bone Marrow Transplant 15: 825–828. 7581076

[20] Remberger M, Ringden O, Hagglund H, Svahn BM, Ljungman P, et al (2013) A high antithymocyte globulin dose increases the risk of relapse after reduced intensity conditioning HSCT with unrelated donors. Clin Transplant 27: E368–374. 23701240 10.1111/ctr.12131

[21] Soudais C, de Villartay JP, Le Deist F, Fischer A, Lisowska-Grospierre B (1993) Independent mutations of the human CD3-epsilon gene resulting in a T cell receptor/CD3 complex immunodeficiency. Nat Genet 3: 77–81. 8490660 10.1038/ng0193-77

[22] Chatenoud L, Bluestone JA (2007) CD3-specific antibodies: a portal to the treatment of autoimmunity. Nat Rev Immunol 7: 622–632. 17641665 10.1038/nri2134

[23] Kaufman A, Herold KC (2009) Anti-CD3 mAbs for treatment of type 1 diabetes. Diabetes Metab Res Rev 25: 302–306. 19319985 10.1002/dmrr.933

[24] Magistrelli G, Jeannin P, Herbault N, Benoit De Coignac A, Gauchat JF, et al (1999) A soluble form of CTLA-4 generated by alternative splicing is expressed by nonstimulated human T cells. European journal of immunology 29: 3596–3602. 10556814 10.1002/(SICI)1521-4141(199911)29:11<3596::AID-IMMU3596>3.0.CO;2-Y

[25] Waterhouse P, Penninger JM, Timms E, Wakeham A, Shahinian A, et al (1995) Lymphoproliferative disorders with early lethality in mice deficient in Ctla-4. Science (New York, N Y ) 270: 985–988. 10.1126/science.270.5238.985 7481803

[26] Ting JP, Trowsdale J (2002) Genetic control of MHC class II expression. Cell 109 Suppl: S21–33 10.1016/s0092-8674(02)00696-7 11983150

[27] Luznik L, Fuchs EJ (2010) High-dose, post-transplantation cyclophosphamide to promote graft-host tolerance after allogeneic hematopoietic stem cell transplantation. Immunol Res 47: 65–77. 20066512 10.1007/s12026-009-8139-0 PMC2892158

[28] Bashey A, Zhang X, Sizemore CA, Manion K, Brown S, et al (2013) T-cell-replete HLA-haploidentical hematopoietic transplantation for hematologic malignancies using post-transplantation cyclophosphamide results in outcomes equivalent to those of contemporaneous HLA-matched related and unrelated donor transplantation. J Clin Oncol 31: 1310–1316. 23423745 10.1200/JCO.2012.44.3523

[29] Luznik L, O’Donnell PV, Fuchs EJ (2012) Post-transplantation cyclophosphamide for tolerance induction in HLA-haploidentical bone marrow transplantation. Semin Oncol 39: 683–693. 23206845 10.1053/j.seminoncol.2012.09.005 PMC3808078

[30] Rosado CJ, Buckle AM, Law RH, Butcher RE, Kan WT, et al (2007) A common fold mediates vertebrate defense and bacterial attack. Science (New York, N Y ) 317: 1548–1551. 10.1126/science.1144706 17717151

[31] Trapani JA (1995) Target cell apoptosis induced by cytotoxic T cells and natural killer cells involves synergy between the pore-forming protein, perforin, and the serine protease, granzyme B. Australian and New Zealand journal of medicine. 25: 793–799. 10.1111/j.1445-5994.1995.tb02883.x 8770355

[32] Lopez JA, Susanto O, Jenkins MR, Lukoyanova N, Sutton VR, et al (2013) Perforin forms transient pores on the target cell plasma membrane to facilitate rapid access of granzymes during killer cell attack. Blood 121: 2659–2668. 23377437 10.1182/blood-2012-07-446146

[33] Martino A, Holmes JHt, Lord JD, Moon JJ, Nelson BH (2001) Stat5 and Sp1 regulate transcription of the cyclin D2 gene in response to IL-2. Journal of Immunology 166: 1723–1729. 10.4049/jimmunol.166.3.1723 11160217

[34] Cao J, Jin Y, Li W, Zhang B, He Y, et al (2013) DNA vaccines targeting the encoded antigens to dendritic cells induce potent antitumor immunity in mice. BMC Immunol 14: 39. 23941509 10.1186/1471-2172-14-39 PMC3751307

[35] Matias BF, de Oliveira TM, Rodrigues CM, Abdalla DR, Montes L, et al (2013) Influence of immunotherapy with autologous dendritic cells on innate and adaptive immune response in cancer. Clin Med Insights Oncol 7: 165–172. 23926442 10.4137/CMO.S12268 PMC3733716

[36] Le DT, Jaffee EM (2012) Regulatory T-cell modulation using cyclophosphamide in vaccine approaches: a current perspective. Cancer Research 72: 3439–3444. 22761338 10.1158/0008-5472.CAN-11-3912 PMC3399042

[37] Wennerberg K, Rossman KL, Der CJ (2005) The Ras superfamily at a glance. J Cell Sci 118: 843–846. 15731001 10.1242/jcs.01660

[38] Goodsell DS (1999) The molecular perspective: the ras oncogene. Stem Cells 17: 235–236. 10437988 10.1002/stem.170235

[39] Bos JL (1989) ras oncogenes in human cancer: a review. Cancer Research 49: 4682–4689. 2547513

[40] El Omari K, Hoosdally SJ, Tuladhar K, Karia D, Vyas P, et al (2011) Structure of the leukemia oncogene LMO2: implications for the assembly of a hematopoietic transcription factor complex. Blood 117: 2146–2156. 21076045 10.1182/blood-2010-07-293357

[41] McCormack MP, Curtis DJ (2010) The thymus under siege: Lmo2 induces precancerous stem cells in a mouse model of T-ALL. Cell Cycle 9: 2267–2268. 20519951 10.4161/cc.9.12.12074

[42] McCormack MP, Young LF, Vasudevan S, de Graaf CA, Codrington R, et al (2010) The Lmo2 oncogene initiates leukemia in mice by inducing thymocyte self-renewal. Science (New York, N Y ) 327: 879–883. 10.1126/science.1182378 20093438

[43] Kearsey SE, Labib K (1998) MCM proteins: evolution, properties, and role in DNA replication. Biochimica et biophysica acta 1398: 113–136. 9689912 10.1016/s0167-4781(98)00033-5

[44] Mantovani A, Bonecchi R, Martinez FO, Galliera E, Perrier P, et al (2003) Tuning of innate immunity and polarized responses by decoy receptors. Int Arch Allergy Immunol 132: 109–115. 14600422 10.1159/000073711

[45] Moschella F, Valentini M, Arico E, Macchia I, Sestili P, et al (2011) Unraveling cancer chemoimmunotherapy mechanisms by gene and protein expression profiling of responses to cyclophosphamide. Cancer Res 71: 3528–3539. 21444678 10.1158/0008-5472.CAN-10-4523

[46] Dale M, Nicklin MJ (1999) Interleukin-1 receptor cluster: gene organization of IL1R2, IL1R1, IL1RL2 (IL-1Rrp2), IL1RL1 (T1/ST2), and IL18R1 (IL-1Rrp) on human chromosome 2q. Genomics 57: 177–179. 10191101 10.1006/geno.1999.5767

[47] Moschella F, Torelli GF, Valentini M, Urbani F, Buccione C, et al (2013) Cyclophosphamide induces a type I interferon-associated sterile inflammatory response signature in cancer patients’ blood cells: implications for cancer chemoimmunotherapy. Clin Cancer Res 19: 4249–4261. 23759676 10.1158/1078-0432.CCR-12-3666

[48] Rosnet O, Schiff C, Pebusque MJ, Marchetto S, Tonnelle C, et al (1993) Human FLT3/FLK2 gene: cDNA cloning and expression in hematopoietic cells. Blood 82: 1110–1119. 8394751

[49] Kuo TC, Tan CT, Chang YW, Hong CC, Lee WJ, et al (2013) Angiopoietin-like protein 1 suppresses SLUG to inhibit cancer cell motility. The Journal of clinical investigation 123: 1082–1095. 23434592 10.1172/JCI64044 PMC3582121

[50] Szabo E, Riffe ME, Steinberg SM, Birrer MJ, Linnoila RI (1996) Altered cJUN expression: an early event in human lung carcinogenesis. Cancer Research 56: 305–315. 8542585

[51] Smith LM, Wise SC, Hendricks DT, Sabichi AL, Bos T, et al (1999) cJun overexpression in MCF-7 breast cancer cells produces a tumorigenic, invasive and hormone resistant phenotype. Oncogene 18: 6063–6070. 10557095 10.1038/sj.onc.1202989

[52] Zhang Y, Pu X, Shi M, Chen L, Song Y, et al (2007) Critical role of c-Jun overexpression in liver metastasis of human breast cancer xenograft model. BMC Cancer 7: 145. 17672916 10.1186/1471-2407-7-145 PMC1959235

[53] Zhang HS, Yan B, Li XB, Fan L, Zhang YF, et al (2012) PAX2 protein induces expression of cyclin D1 through activating AP-1 protein and promotes proliferation of colon cancer cells. Journal of Biological Chemistry 287: 44164–44172. 23135283 10.1074/jbc.M112.401521 PMC3531732

[54] Vleugel MM, Greijer AE, Bos R, van der Wall E, van Diest PJ (2006) c-Jun activation is associated with proliferation and angiogenesis in invasive breast cancer. Hum Pathol 37: 668–674. 16733206 10.1016/j.humpath.2006.01.022

[55] Jiao X, Katiyar S, Willmarth NE, Liu M, Ma X, et al (2010) c-Jun induces mammary epithelial cellular invasion and breast cancer stem cell expansion. J Biol Chem 285: 8218–8226. 20053993 10.1074/jbc.M110.100792 PMC2832973

[56] Wisdom R, Johnson RS, Moore C (1999) c-Jun regulates cell cycle progression and apoptosis by distinct mechanisms. The EMBO journal 18: 188–197. 9878062 10.1093/emboj/18.1.188 PMC1171114

[57] Schreiber M, Kolbus A, Piu F, Szabowski A, Mohle-Steinlein U, et al (1999) Control of cell cycle progression by c-Jun is p53 dependent. Genes & development 13: 607–619. 10072388 10.1101/gad.13.5.607 PMC316508

[58] Kollmann K, Heller G, Ott RG, Scheicher R, Zebedin-Brandl E, et al (2011) c-JUN promotes BCR-ABL-induced lymphoid leukemia by inhibiting methylation of the 5′ region of Cdk6. Blood 117: 4065–4075. 21300982 10.1182/blood-2010-07-299644

[59] Ritke MK, Bergoltz VV, Allan WP, Yalowich JC (1994) Increased c-jun/AP-1 levels in etoposide-resistant human leukemia K562 cells. Biochem Pharmacol 48: 525–533. 8068039 10.1016/0006-2952(94)90282-8

[60] Rubin E, Kharbanda S, Gunji H, Weichselbaum R, Kufe D (1992) cis-Diamminedichloroplatinum(II) induces c-jun expression in human myeloid leukemia cells: potential involvement of a protein kinase C-dependent signaling pathway. Cancer Res 52: 878–882. 1737349

[61] Zhou J, Gupta K, Yao J, Ye K, Panda D, et al (2002) Paclitaxel-resistant human ovarian cancer cells undergo c-Jun NH2-terminal kinase-mediated apoptosis in response to noscapine. J Biol Chem 277: 39777–39785. 12183452 10.1074/jbc.M203927200

[62] Gurzov EN, Bakiri L, Alfaro JM, Wagner EF, Izquierdo M (2008) Targeting c-Jun and JunB proteins as potential anticancer cell therapy. Oncogene 27: 641–652. 17667939 10.1038/sj.onc.1210690

[63] Xia Y, Yang W, Bu W, Ji H, Zhao X, et al (2013) Differential Regulation of c-Jun Protein Plays an Instrumental Role in Chemoresistance of Cancer Cells. Journal of Biological Chemistry 288: 19321–19329. 23678002 10.1074/jbc.M113.475442 PMC3707636

