# Correction: Cyclophosphamide Alters the Gene Expression Profile in Patients Treated with High Doses Prior to Stem Cell Transplantation

**DOI:** 10.1371/journal.pone.0093239

**Published:** 2014-03-18

**Authors:** The PLOS ONE Staff

The affiliation of the fourth author is incorrect. Parvaneh Afsharian is affiliated with: Pharmacogenetics Group, Department of Genetics, Reproductive Biomedicine Research Center, Royan Institute for Reproductive Biomedicine, ACECR, Tehran, Iran.
